# Balancing memory in sleep: firing barrages as a circuit breaker for reactivation

**DOI:** 10.1038/s41392-024-02057-y

**Published:** 2024-11-22

**Authors:** Hayder Amin

**Affiliations:** 1 Group of “Biohybrid Neuroelectronics”, German Center for Neurodegenerative Diseases (DZNE), Dresden, Germany; 2 TU Dresden, Faculty of Medicine Carl Gustav Carus, Dresden, Germany

**Keywords:** Cellular neuroscience, Bioinformatics, Diseases of the nervous system

In their recent study published in *Science*, Karaba and colleagues reported a novel mechanism within the hippocampus that balances memory reactivation during sleep.^1^ The authors demonstrated that a distinct network event termed the barrage of action potentials (BARRs) counteracts the heightened neural activity induced by sharp-wave ripples (SWRs).^2^ This study reveals how BARRs, originating from CA2 pyramidal neurons, modulate inhibitory interneurons to suppress SWR-driven reactivation, ensuring network stability during non-rapid eye movement (NREM) sleep.^2^

**Fig. 1:**
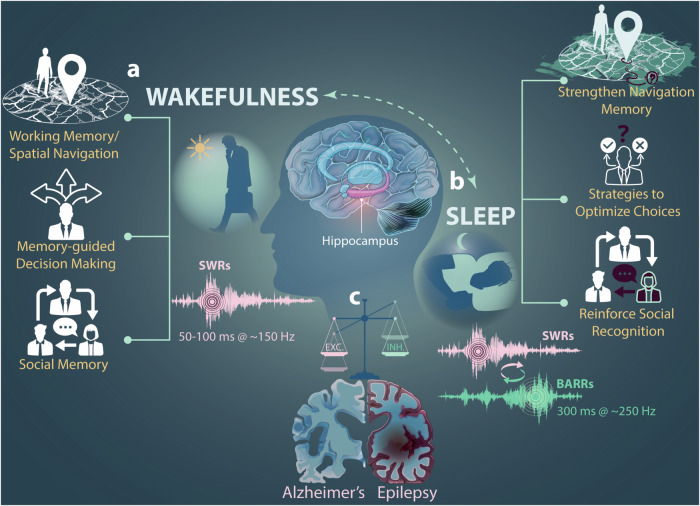
Wakefulness and Sleep: Neural Patterns for Memory Encoding and Consolidation. **a** In a wakeful state, the brain encodes diverse real-world experiences such as learning tasks, decision-making strategies, and social interactions. These experiences engage hippocampal circuits, driving key activity patterns like sharp-wave ripples (SWRs), theta waves, and gamma oscillations. The hippocampus encodes these experiences through synaptic plasticity, with specific activity bursts in time and frequency supporting learning and memory encoding. **b** During non-REM sleep, the brain consolidates these wakeful experiences. The hippocampus replays and reactivates the same neurons engaged during wakefulness, with SWRs driving the reactivation. Importantly, BARRs provide inhibitory regulation, ensuring balanced reactivation and preventing excessive excitation. This balance counteracts SWRs, maintaining network stability and enabling effective memory consolidation. **c** The balance between excitation and inhibition (EXC./INH.) is essential for computational efficiency and network stability during both wakeful learning and sleep-dependent consolidation. In conditions like epilepsy, a lack of inhibition leads to excessive synchrony, resulting in seizures and disrupted memory processes. In Alzheimer’s disease, early-stage network hyperactivity and impaired inhibition hinder memory consolidation, contributing to cognitive decline. Disruptions in EXC./INH. balance in these disorders lead to memory deficits, highlighting potential therapeutic targets for regulating memory consolidation processes. Waveforms of SWRs and BARRs are representative and do not depict actual data

Consolidating the day’s experiences into lasting memories requires more than just waking attention—the brain works behind the scenes during sleep to solidify and balance those experiences (Fig. 1). Memory consolidation is a critical function of the hippocampus, largely dependent on neural reactivation during NREM sleep. During this phase, SWRs are the dominant form of activity, with neurons involved in recent experiences showing increased firing rates. But how does the brain prevent reactivation from becoming excessive, avoiding a shift from stable memory formation to network instability? The answer lies in mechanisms like BARRs, which fine-tune neural activity to avoid overexcitation, offering a new perspective on how the hippocampus maintains stability and homeostasis during sleep for memory consolidation.

*Karaba* et al. uncovered BARRs, a long-duration network event originating from CA2 pyramidal cells. Unlike SWRs (~50 ms), which are short synchronized bursts, BARRs last up to 300 milliseconds and are anti-correlated with SWRs, alternating in time. BARRs inhibit the reactivation of CA1 neurons involved in learning, preventing prolonged excitation. The study highlights the role of cholecystokinin-expressing (CCK+) basket cells, driven by CA2 neurons during BARRs, which selectively inhibit the same CA1 neurons activated during SWRs. This balance between SWR-induced excitation and BARR-mediated inhibition ensures neuronal firing returns to baseline after learning. Optogenetic silencing of CCK+ cells increased CA1 synchrony and firing rates, impairing memory consolidation, showing that the interplay between SWRs and BARRs is essential for both memory enhancement and the functional stability of the hippocampal network.

The study highlighted the distinct timing and recruitment patterns of SWRs and BARRs. SWRs typically occur across multiple hippocampal subregions, synchronizing neurons involved in encoding recent experiences. In contrast, BARRs are localized events that involve prolonged firing of CA2 pyramidal neurons and selective activation of CCK+ interneurons. These interneurons then inhibit CA1 pyramidal cells, particularly those reactivated during SWRs. Interestingly, the researchers found that the neurons most affected by BARRs were those with the strongest reactivation during learning-related tasks. This suggests that BARRs play a critical role in downregulating the activity of learning-involved neurons, preventing excessive synchrony that could destabilize the network. Recordings from CA1, CA2, and CA3 hippocampus regions in mice performing memory tasks showed that the frequency of SWRs and BARRs increased during sleep following learning. However, the two events were anti-correlated, with BARRs occurring after SWRs to suppress the heightened activity of CA1 neurons.

The discovery of BARRs adds a new dimension to our understanding of memory consolidation. SWRs have been regarded as the primary driver of sleep-dependent memory consolidation, with studies linking their activity to improved memory performance and disruption leading to impairment. The identification of BARRs introduces a critical inhibitory mechanism that counterbalances the excitatory effects of SWRs, aligning with the *synaptic homeostasis hypothesis*, which suggests that sleep downscales synaptic connections that have been potentiated during wakefulness (Fig. 1). BARRs likely act as a homeostatic mechanism, preventing runaway excitation by inhibiting CA1 neurons after SWR reactivation, thus restoring balance in the hippocampus and ensuring memory consolidation proceeds without hyperactivity. The *Memory Allocation Hypothesis* adds another layer of insight, positing that highly excitable neurons are preferentially recruited during learning. BARRs complement this by selectively inhibiting these reactivated neurons during SWRs, preventing overstimulation and stabilizing the network. This fine-tuning of neural excitability by BARRs not only supports balanced memory consolidation but may enhance computational efficiency within the hippocampal circuitry, much like balanced networks that optimize coding precision and minimize firing variability, ensuring robust information processing during memory consolidation.^3^

The study further shows that memory consolidation relies on the precise interplay between SWRs and BARRs. Disrupting BARRs increases CA1 synchrony, impairs memory, and highlights the importance of both events for proper memory consolidation.

The identification of BARRs has important implications for understanding the neural mechanisms underlying memory-related disorders.^4^ Conditions such as epilepsy and Alzheimer’s disease (AD) are associated with disruptions in neural synchrony and memory consolidation. The discovery that BARRs balance memory reactivation suggests that dysregulation of this mechanism could contribute to cognitive deficits in these disorders (Fig. 1). In epilepsy, excessive synchrony in the hippocampus is a hallmark of seizure activity. Understanding how BARRs prevent such synchrony during sleep could inform therapeutic strategies to restore the balance between excitatory and inhibitory activity. Similarly, in AD, early-stage network hyperactivity is tied to cognitive decline due to the loss of compensatory mechanisms. By regulating neuronal excitability and preventing hyperactivity, BARRs may protect against the neuronal dysfunction seen in AD, offering potential for enhancing memory and slowing disease progression. It remains unclear whether the occurrence of BARRs is modulated by the strength of learning or by other factors, such as the state of the hippocampal network. Additionally, investigating how BARRs interact with different forms of hippocampal oscillatory activity, such as theta and gamma rhythms, could provide deeper insights into the coordination of memory processes during sleep.

Karaba et al.’s discovery of BARRs as a counterbalance to SWRs during sleep marks a key advancement in memory research, revealing how the brain prevents excessive synchrony during memory consolidation. The interplay between SWRs and BARRs underscores the complexity of sleep-dependent neural processes and highlights the importance of both excitatory and inhibitory mechanisms in maintaining hippocampal stability. Notably, rich experiences that reshape hippocampal network dynamics and connectivity^5^ may similarly influence BARR regulation, adding a layer of adaptability to hippocampal function. Given this adaptability, could the modulation of BARRs be a key to preventing the early hyperactivity seen in neurodegenerative diseases, and might it offer new avenues for therapeutic intervention? Answering these questions will be critical as research on BARRs progresses in the quest to understand and treat memory-related disorders.
